# Search for Expectancy-Inconsistent Information Reduces Uncertainty Better: The Role of Cognitive Capacity

**DOI:** 10.3389/fpsyg.2016.00395

**Published:** 2016-03-22

**Authors:** Paweł Strojny, Małgorzata Kossowska, Agnieszka Strojny

**Affiliations:** ^1^Social Psychology Unit, Department of Psychology, Jagiellonian UniversityKraków, Poland; ^2^Neurocognitive Psychology Unit, Department of Psychology, Pedagogical University of CracowKraków, Poland

**Keywords:** need for closure, uncertainty reduction, cognitive strategy, cognitive capacity, knowledge formation, information processing, confirmation bias

## Abstract

Motivation and cognitive capacity are key factors in people’s everyday struggle with uncertainty. However, the exact nature of their interplay in various contexts still needs to be revealed. The presented paper reports on two experimental studies which aimed to examine the joint consequences of motivational and cognitive factors for preferences regarding incomplete information expansion. In Study 1 we demonstrate the interactional effect of motivation and cognitive capacity on information preference. High need for closure resulted in a stronger relative preference for expectancy-inconsistent information among non-depleted individuals, but the opposite among cognitively depleted ones. This effect was explained by the different informative value of questions in comparison to affirmative sentences and the potential possibility of assimilation of new information if it contradicts prior knowledge. In Study 2 we further investigated the obtained effect, showing that not only questions but also other kinds of incomplete information are subject to the same dependency. Our results support the expectation that, in face of incomplete information, motivation toward closure may be fulfilled efficiently by focusing on expectancy-inconsistent pieces of data. We discuss the obtained effect in the context of previous assumptions that high need for closure results in a simple processing style, advocating a more complex approach based on the character of the provided information.

## Introduction

Although some degree of uncertainty in our lives may be stimulating, too much of it could be uncomfortable, particularly if it concerns self-uncertainty or uncertainty about matters that directly reflect on one’s self-concept (e.g., Frenkel-Brunswik, 1949; Gudykunst, 1985; Levine, 1985; Sorrentino and Short, 1986; Kruglanski, 2004; Mendes et al., 2007). Generally, people strive to reduce feelings of uncertainty about themselves, their social world and their place within it: they like to know who they are and how to behave, and who others are and how they might behave. But, there are also differences between individuals in terms of tolerance of uncertainty and thus the strategies used to cope with it (Frenkel-Brunswik, 1949; Smock, 1955; Rokeach, 1960; Bunder, 1962; Rydell and Rosen, 1966; Kagan, 1972; Sorrentino and Short, 1986; Neuberg and Newsom, 1993; Webster and Kruglanski, 1994; Cialdini et al., 1995; Bar-Tal et al., 1997). One of these characteristics is the need for closure, described as the tendency to reduce the feeling of discomfort experienced in the face of cognitive uncertainty through the rapid formulation and brief validation of a hypothesis (Webster and Kruglanski, 1994). Some research has demonstrated that focusing on expectancy-consistent information allows individuals with high need for closure to navigate a stream of ambiguous information, most of which challenges their *a priori* expectancy (Driscoll et al., 1991; Dijksterhuis et al., 1996; Macrae and Bodenhausen, 2000; Skowronski et al., 2013). However, there are some findings showing that higher need for closure may sometimes lead to reducing uncertainty via processing expectancy–inconsistent information (Sherman et al., 1998, 2004; Kemmelmeier, 2015). Thus, in our study we focus on the relationship between need for closure and preference for expectancy-inconsistent information as a strategy to reduce uncertainty. As previous research focused on searching for information in the form of affirmative sentences (i.e., individuals are provided with complete, ready to process information, e.g., “Took money he/she found in the street to a police station.”), we focus on situations in which individuals search for information to be acquired (i.e., unanswered questions, incomplete information to be extended; e.g., “Did he/she take money he/she found in the street to a police station?”). Since a person faced with incomplete information does not know in advance whether the full meaning of this information will be consistent or inconsistent with the hypothesis under consideration, increased need for closure relates to a stronger tendency to prefer expectancy-inconsistent questions.

In addition, processing of information which contradicts pre-existing beliefs is effortful and needs cognitive capacity (Piaget, 1928; Hastie and Kumar, 1979; Crocker et al., 1983; Stangor and McMillan, 1992). Thus, we predicted that the preference for expectancy-inconsistent information provided in incomplete form (i.e., a question to be asked), would become a preference for expectancy-consistent information when cognitive capacity is depleted.

### Need for Closure and Preferences for Expectancy-Consistent and -Inconsistent Information as Different Strategies of Uncertainty Reduction

Some research has demonstrated that need for closure is usually related to reducing uncertainty via restricted use of relevant cues or crude categories, which often leads to biased judgments, stereotyping or prejudice (e.g., Driscoll et al., 1991; Shah et al., 1998; Kruglanski et al., 2002, 2009). Moreover, it has been shown that need for closure leads to actively searching for information that supports preexisting views and/or avoiding disconfirming information, be it in the domain of attitudes, beliefs, or stereotypes. For example, Dijksterhuis et al. (1996) demonstrated that need for closure increased the ability to memorize stereotype-consistent information in order to confirm closure regarding the characteristics of the target group the participants read about at the beginning of the procedure. As a consequence, need for closure positively correlated with more stereotypical evaluation of the target group, and negatively with preference for stereotype-inconsistent information. Also, Kossowska and Bar-Tal (2013) demonstrated that when participants expected to successfully achieve closure, higher need for closure led them to recall relatively less stereotype-inconsistent information, while there was no statistically significant difference in the amount of stereotype-consistent information recalled. These phenomena may be viewed as a specific cases of confirmation bias, understood as seeking and interpreting evidence in favor of beliefs, expectations or hypothesis (Nickerson, 1998). However, we decided to use the term “expectancy-consistent information search” in the current paper for two reasons. Firstly, the term “confirmation bias” carries the presumption that such behavior is somehow wrong, but in fact, it could be efficient both in terms of achieving the desired state of closure (Kruglanski and Mayseless, 1988) and in terms of real-life decision making (e.g., Klayman and Ha, 1987). Secondly, as Nickerson (1998, p. 175) stated, conformation bias is “a ubiquitous phenomenon in many guises”, thus using this term in our research which was not designed to study this phenomenon may lead to overgeneralization. In conclusion, we use the term “search for expectancy-consistent information” (as the complement of “search for expectancy-inconsistent information” which we actually focus on) in the entire text, but some of the readers may find our results interesting also in the broader context of confirmation bias research.

Thus, it seems that when individuals have just formulated a hypothesis, need for closure is manifested in strong avoidance or even a tendency to ignore the new, contradicting information because it endangers the comfortable state of closure (Roets et al., 2015 for review). As preference-consistent information reduces uncertainty, evokes positive associations, and has positive implications for the self, refuting inconsistent information is the most effective way for individuals high in need for closure to achieve an enduring and stable uncertainty reduction.

Thus, for high in need for closure individuals, focusing on what is already known is much more likely to reduce subjective feelings of uncertainty and affirm existing beliefs. However, when the majority of the encountered evidence is not supportive of one’s expectancy, uncertainty is not reduced and existing beliefs are not enhanced when participants dwell on expectancy-consistent information (cf. Sherman et al., 1998, 2004; Kemmelmeier, 2015). Rather, in order to affirm existing beliefs, need for closure may lead to paying attention to information that does challenge the previously formulated expectancy, possibly because thinking about such information might allow it to be reconciled with the expectancy. For instance, if high in need for closure individuals expect Mark to be a highly competent worker, but learn that he is not very reliable in his work, they might consider that he was bored and under-challenged, and that he was wasting his time in the company. Thus, paying attention to expectancy-inconsistent information might be driven as much by the motivation to reduce ambiguity and affirm preexisting beliefs as is attending to expectancy-consistent information. In our study we aim to further explore the relationship between need for closure and preference for expectancy-inconsistent information as a strategy to reduce uncertainty.

### Preference for Consistency vs. Inconsistency Depends on the Way Information is Provided

The meaning of lack of closure may be inferred from the definition of need for cognitive closure: if the need for closure is “the desire for a firm answer to a question and an aversion toward ambiguity” (Kruglanski and Webster, 1996, p. 264), lack of closure occurs in situations in which at least one question remains unanswered, therefore the intensity of this lack perhaps grows in line with the number of unanswered questions. As a consequence, high in need for closure individuals confronted with any unclear issue (e.g., assessing a person’s characteristics) quickly adopt the first hypothesis (if one has been suggested; see Kemmelmeier, 2015) and tend to ignore contradictory information (e.g., Dijksterhuis et al., 1996; Kossowska and Bar-Tal, 2013). It is widely agreed that acquisition and integration of information is necessary before knowledge can be formulated (e.g., Popper, 1959; Kruglanski et al., 2009). The term ‘information’ defined as “facts provided or learned about something or someone” (e.g., Oxford’s advanced learner’s dictionary, n.d.) is crucial in this reasoning. Since all incomplete data, such as an unanswered question or an excerpt of an opinion, does not provide undeniable information, it differs in epistemic consequences from affirmative sentences. The legitimate conclusions of reading the sentences “He/she paid someone to write an M.A. thesis for him/her.” and “Did he/she pay someone to write an M.A. thesis for him/her?” about a person who seems to be honest are different (compare with, e.g., Kossowska and Bar-Tal, 2013). Being confronted with the fact in the former sentence denies the preliminary hypothesis and may result in contesting, if not altogether ignoring new information, especially by people who appreciate certainty (i.e., high in need for closure individuals). In contrast, posing the latter sentence only suggests the other possibility and therefore cries out for an answer which has a high likelihood of being negative in the light of the previously formulated hypothesis (and even if it is positive, it can be reconciled). This tendency should increase with increased need for closure, since increased need for closure is reflected in intolerance of ambiguity (e.g., Roets and Van Hiel, 2008).

Since we do not limit our reasoning solely to questions, but instead treat this mode of sentence as an exemplar of a wider category of ‘information to be acquired’ (in contrast to complete information used in the majority of previous studies; but see Kruglanski and Mayseless, 1988), we claim that the consequences of other incomplete information for the epistemic process should be similar to those of the question. Obviously a question and an incomplete excerpt of an opinion differs formally (e.g., “Did he/she pay someone to write an M.A. thesis for him/her?” vs. “I suspect he/she paid someone to write an M.A. thesis for him/her because…”) but, in contrast to complete information, both forms do not provide certain knowledge and rather emphasize the uncertainty about a given issue. Consequently, we expect both forms to produce similar effects.

Thus we predict that when individuals are instructed to select information to be acquired (e.g., “Which question do you prefer to ask to find out…”), need for closure is linked to a preference for expectancy-inconsistent data. We expected that increased need for closure leads to less tolerance for the uncertainty evoked by this kind of incomplete information. As a consequence, a higher need for closure is linked to finding an answer and either confirming a hypothesis (when the answer turns out to be negative, i.e., expectancy-consistent) or reconciling newly acquired information (when the answer turns out to be positive).

### Preference for Inconsistency Requires Cognitive Capacity

Researchers have argued for a long time that inconsistent information prompts cognitive efforts to reconcile it with preexisting knowledge (Piaget, 1928; Hastie and Kumar, 1979; Crocker et al., 1983; Stangor and McMillan, 1992). In order to do that, individuals have to engage in a process of assimilation “to transform perceptions until they are identical to one’s own thought, i.e., with previous schemas” (Piaget, 1928, p. 174). The assimilation of information pieces is assumed to be cognitively effortful as it consists of deliberate and controlled information processing (Piaget, 1928; Hastie and Kumar, 1979; Crocker et al., 1983; Stangor and McMillan, 1992; Kemmelmeier, 2015). For example, Kemmelmeier argues that “such [expectancy-inconsistent] information prompts efforts to reconcile it with one’s expectancy, with the resulting cognitive elaboration making expectancy-inconsistent information more memorable than expectancy-consistent information” (Kemmelmeier, 2015, p. 3; see also Macrae et al., 1993). Also, researchers on the dual-type model (e.g., Petty and Cacioppo, 1986; Chaiken et al., 1989) and the unified model (Erb et al., 2003) of information processing indicate that deliberative information processing requires certain cognitive capacity (e.g., Kruglanski and Gigerenzer, 2011; Evans and Stanovich, 2013). For example, Evans and Stanovich (2013) claim that this kind of cognitive process (i.e., ‘Type 2 process’) requires working memory capacity and is typically correlated with capacity limitations and cognitive ability, thus an individual lacking cognitive capacities is virtually unable to perform it. Also, Ditto and Lopez (1992) argued that the tendency to choose preference-consistent information over preference-inconsistent information is less likely to initiate effortful cognitive analysis. From this perspective, rather than actively working to construct justifications for preference-consistent information, people often unthinkingly accept information they want to believe “at face value”. Information inconsistent with a preferred judgment conclusion is, on the other hand, more likely to initiate an effortful cognitive appraisal in which alternative explanations for the unwanted information are likely to be considered, producing uncertainty regarding the validity of the information.

Moreover, Kruglanski and Gigerenzer (2011, p. 103) stated that in the situation of capacity depletion “the individual may be loathe to apply rules^1^ whose implementation requires laborious computational analyses” and, as a consequence, utilizes only relatively simple judgmental rules and finds it difficult to assess their value. Thus high cognitive capacity is a prerequisite for engaging in the process of information reconciliation, otherwise it would be more efficient to discard all awkward questions in order to avoid confronting expectancy-inconsistent answers that cannot be elaborated due to cognitive ability deficit.

Since increased need for closure is manifested in intense experience of uncertainty in the face of a lack of closure (Roets and Van Hiel, 2008), it leads to the formulation of as much cohesive knowledge as possible. In the face of an ambiguous information set, the increased need for closure sensitizes the subject to expectancy-inconsistent information because it prompts special attention in order to reconcile it with already learnt information (e.g., Hastie and Kumar, 1979; Stangor and McMillan, 1992; Strojny, unpublished doctoral dissertation), which in turn results in better memory for this information (Macrae et al., 1993; Kemmelmeier, 2015). If this way of reasoning is valid, cognitive capacity should be a necessary condition for a preference for expectancy-inconsistent information. Otherwise, if cognitive capacity is compromised, a higher need for closure should increase preference for expectancy-consistent over inconsistent information as the inability to reconcile expectancy-inconsistent information is due to cognitive ability deficit.

On the other hand, individuals low in cognitive capacity are not only incapable of successfully processing inconsistent information in order to reconcile it with previous knowledge, but they may be more likely to prefer expectancy-consistent data due to their general propensity to behave reflectively. Previous research has demonstrated that impulsive actions are more dominant among people lacking the cognitive ability necessary to override them (Engle, 2002; Barrett et al., 2004). Hofmann et al. (2008) showed that low-working memory capacity individuals were more likely to manifest behavior consistent with their dispositions than inconsistent behavior. Macrae et al. (1993) also demonstrated that whereas perceivers displayed preferential recall for stereotype-inconsistent information under low processing load (higher cognitive capacity), this switched to a preference for consistent information as task demands increased (lower cognitive capacity). Thus, low in cognitive capacity people may be more likely to select expectancy-consistent incomplete information merely because of their ostensible meaning, even if the question (or a fragment of information) does not necessarily mean what it asks, it may do so in the eyes of a cognitively depleted person.

Thus, bearing in mind that cognitive depletion prevents the effortful process of information assimilation, we expect that when participants are depleted, a higher need for closure leads to a preference for more expectancy-consistent data.

We formulated two hypotheses:

Hypothesis 1: In the face of incomplete information, the level of need for closure correlates positively with the preference for expectancy-inconsistent information among individuals with relatively high cognitive capacity.
Hypothesis 2: In the face of incomplete information, the level of need for closure correlates negatively with the preference for expectancy-inconsistent information among individuals with relatively low cognitive capacity.

### Overview of the Study

Although the results of much research leads to the conclusion that for high need for closure individuals the most preferred way of uncertainty reduction is to search for expectancy-consistent information, we think that this is not always true. Thus, we predict that when individuals are instructed to select information to be acquired (e.g., “Which question do you prefer to ask to find out…”), need for closure is linked to a preference for expectancy-inconsistent information. However, when participants are cognitively depleted, the higher need for closure leads to a preference for more expectancy-consistent data.

We tested these hypotheses in two studies. We operationalized *need for closure* as a level on a scale (Webster and Kruglanski, 1994). By the term *cognitive capacity* we meant an individual’s limited and taxable pool of cognitive resources to process information (Roets et al., 2013). We experimentally depleted that pool with a character repetition task (Gilbert and Osborn, 1989; Gilbert and Hixon, 1991) as this manipulation was usually applied in previous research on epistemic processes (e.g., Bar-Tal et al., 1999, Study 3; Roets and Van Hiel, 2007, Study 3).

In Study 1 we measured preference for expectancy-consistent compared to expectancy-inconsistent information with a version of a classic task (Snyder and Swann, 1978). Kruglanski and Mayseless (1988) previously used by Kossowska and Bar-Tal (2013), with modification of sentence mode. Specifically, we replaced affirmative sentences with interrogative ones while keeping the content of the former unchanged. In Study 2 we altered the form of presentation information to be acquired once again to make sure that the key factor of the obtained results was not the interrogative sentence mode but rather the inconclusive way in which the information was provided. This time we used brief fragments of information which may be acquired.

## Study 1

### Method

#### Participants

The study included 67 high school students aged 17–20 years (*M* = 17.63, *SD* = 0.62), 61 of whom were women. The gender disproportion was not intended and resulted from unexpected uneven interest in participating in the experiment between both genders. Two participants failed to successfully perform the cognitive capacity depletion task and were excluded from the analysis, which left us with a sample of 65 participants (*M* = 17.62, *SD* = 0.62, 59 women). In exchange for their participation, each participant could win one of two tickets in a lottery (each worth approximately $8).

#### Materials

##### Need for closure

To assess individual levels of need for closure, we used the Need for Cognitive Closure Scale (Webster and Kruglanski, 1994). Due to controversy regarding the nature of the Decisiveness subscale (see Roets and Van Hiel, 2007) we excluded items related to this subscale from the scale. Finally, we analyzed the results obtained with 27 items belonging to four subscales (Preference for order, Preference for predictability, Intolerance of ambiguity, Closed-mindedness) with acceptable reliability (Cronbach’s α = 0.69, *M* = 3.77, *SD* = 0.40).

##### Cognitive capacity depletion

In the low cognitive capacity condition, a sequence of eight characters (e.g., 9KWELB1P) had to be memorized, whereas in the high cognitive capacity condition, no character had to be memorized (for a similar manipulation, see e.g., Roets et al., 2013). The sequence was administered directly before the start of the main task and subjects were informed that they must keep it in mind throughout and that at the end of the procedure their memory of it would be tested.

##### Preference for expectancy-consistent vs. –inconsistent information

The dependent variable was measured with a task similar to the one used in the study by Bar-Tal and Kossowska (2010 Study 3, Kossowska and Bar-Tal, 2013, Study 1). Participants were asked to imagine that they had just met a person who at a first glance appears to be dishonest. This information was intended to suggest the initial hypothesis about the characteristics of the target person. Afterward participants were presented with fifteen questions regarding the preliminary hypothesis; five consistent (e.g., “Did he cheat during the exam?”), five inconsistent (e.g., “Did he return the money found on the street?”) and five irrelevant (e.g., “Does he read the daily newspaper?”). They were asked to assess (on 7-point scales) the extent to which each of the questions would be useful to determine whether the target person was actually dishonest. We calculated index of preference for inconsistency as the proportion of preference for inconsistent questions to the preference for all relevant questions assessed by participants, multiplied by 100. The greater the score, the stronger the relative inclination toward expectancy-inconsistent questions.

##### Manipulation check

Two questions were used in order to check the effectiveness of cognitive capacity depletion: “How burdensome was the task?” and “How hard was the task?”. Questions were answered on a scales ranging from 1 to 6 (Cronbach’s α = 0.80). At the end of the study the actual memory of the strings was tested to eliminate participants who did not understand or failed to follow the instructions. The minimum performance was set to at least one correctly recalled character in the correct position. Results of people who did not meet this criterion were excluded from the analysis to avoid the risk of analyzing the results of subjects who were not actually depleted^2^.

#### Procedure

Participants were informed that the study concerned the relationship between cognitive style and judgment during recruitment. After obtaining informed consent, they were given materials printed on paper. They were first asked to complete a need for closure questionnaire. Afterward, they were randomly assigned to one of the two conditions: if a participant belonged to the experimental group, they received a random sequence of characters to be remembered in print. They gave their sheets back before starting the main task. All the participants were then given another piece of paper with instructions to imagine they had just met a person who, at first glance, appears to be dishonest and their task was to assess the answers to which of the presented questions would be useful to verify if this person is indeed dishonest. The last part contained manipulation check questions and a sequence memory test. Finally, participants were debriefed and thanked.

### Results and Discussion

#### Manipulation Check and Preliminary Analyses

To check whether the manipulation was effective we used two questions concerning the onerousness and difficulty of the task. The experimental group reported a slightly higher capacity depletion (*M* = 2.32, *SD* = 1.11) than the control group (*M* = 2.12; *SD* = 0.99), but the difference was not significant [*t* (63) = 0.78, *p* = 0.44, *d* = 0.19). Thus, the manipulation may be assessed to not be effective enough. Similar results were found in several previous studies (e.g., Bar-Tal et al., 1999, Study 3). Researchers speculated that some participants could merge the difficulty of the capacity depletion task and the main task because the manipulation was followed by the main task, which in turn was followed by the manipulation check, which could also be the case in the current study. Consequently, it was decided to carry out the planned analysis, including the manipulation check as covariate. We also checked the results of secondary task itself, they indicated that the participants performed, on average, very well (*M* = 6.64, *SD* = 2.37), two participants did not recall one single character correctly and was excluded from further analysis.

#### Need for Closure and Preference for Expectancy-Inconsistent Information

Neither cognitive capacity depletion (*M*_contr_ = 54.1, *SD* = 7.34, *M*_exp_ = 51.9, *SD* = 7.14, *t*(63) = 1.28, *p* = 0.21, *d* = 0.3) nor need for closure (*b* = –0.015, *t*(63) = –0.18, *p* = 0.86, 95% CI: [–0.18, 0.15]) affected the preference for expectancy-inconsistent information on their own.

#### Need for Closure and Cognitive Capacity Joint Effect on the Preference for Expectancy-Inconsistent Questions

**FIGURE 1:**
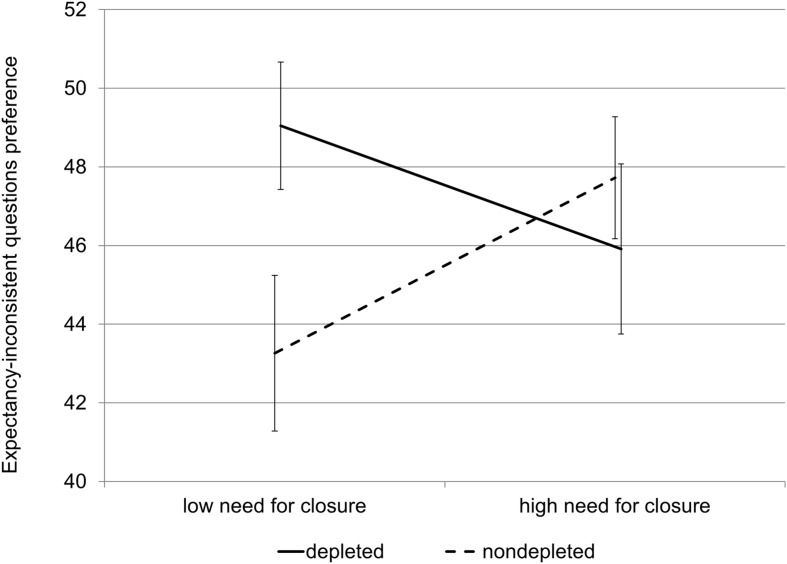
**Interaction effect between need for closure and cognitive capacity on expectancy-inconsistent questions preference (Study 1).** Error bars represent standard errors.

We used PROCESS to test the interactional effect of cognitive capacity depletion on the relationship between need for closure and preference for expectancy-inconsistent information (Hayes, 2013, model 1). The influence of gender and age on the interaction was inspected, but it turned out to be statistically insignificant and these variables were removed from the analysis. We found a statistically significant interaction between need for closure and cognitive capacity (*b* = –0.35, β = –1.91, *SE* = 0.17, *p* < 0.05, 95% CI [–0.69, –0.01]). The results are shown in **Figure 1.** Further analysis of the interaction showed that need for closure correlated positively with the index of expectancy-inconsistent questions preference in the control condition (*b* = –0.20, β = –2.23, SE = 0.11, *p* = 0.08, 95% CI: [–0.43, 0.03]). In the low capacity condition the correlation tended to be the opposite and became statistically insignificant (*b* = 0.14, β = 1.56, *SE* = 0.12, *p* = 0.19, 95% CI: [–0.11, 0.39]).

The results of Study 1 are in line with our expectations, but they did not reach significance level. Thus we cannot make an unambiguous inference about the nature of the joint influence of need for closure and cognitive capacity on expectancy-inconsistent incomplete information. The possible explanation is that, using the method utilized in Study 1 (and before by Bar-Tal et al., 1997, 1999; Bar-Tal and Kossowska, 2010; Kossowska and Bar-Tal, 2013), it is not possible to be sure whether the single sentence designed to evoke preliminary hypothesis about the target person (“*At the first glance he seems dishonest*”) fulfilled its role. For some participants, such information is probably not a sufficiently strong argument in favor of the initial hypothesis about the target person. If this was true, it could have reduced the size of the effect. We decided to alter the method used in Study 2 in order to test the reliability and increase the internal validity of the obtained results.

## Study 2

Study 1 provided the reasons to argue that need for closure leads to searching for expectancy-inconsistent information supplementation in order to achieve certainty. According to our predictions, the effect occurred only among people in the control group with relatively high cognitive capacity, the necessary condition of successful information reconciliation. The purpose of Study 2 was to verify the previously obtained effect with the use of a different form of information to be acquired. Because of the doubts regarding the previously used task, it was decided to use a different method developed by Frey (e.g., Frey, 1986; Jonas et al., 2003; Fischer et al., 2005, 2011). According to this method, participants were asked to decide whether a manager’s contract should be extended or not. They were provided with shortened opinions about the manager. They task was to select opinions which they wanted to read in the full version. The main advantage of the new method was that it enabled us to increase the generality of the results, due to replacement of questions with brief fragments of opinions which were expanded if selected. Moreover, because the method used in Study 2 allows participants to formulate preliminary impression on their own, we were able to eliminate the doubts about the initial hypothesis induction mentioned in the discussion in Study 1.

### Method

#### Participants

High school students aged 18–20 years took part in the procedure (*N = 94, M* = 18.65, *SD* = 0.66) including 72 women, 20 men, and 2 subjects who did not specify their gender. One participant withdrew before the end of the procedure. During preliminary analysis the results of 20 participants were eliminated due to the previously determined reasons described below (cognitive capacity load task failure or inability to formulate the preliminary hypothesis). We conducted analysis of 74 subjects aged 18–20 (*M* = 18.67, *SD* = 0.67): 56 women, 17 men, and one participant with unspecified gender. Subjects were invited to participate in the study after obtaining the consent of the school head and teacher present during the procedure. All participants were unaware of the purpose of the research and hypotheses. The participation in a lottery for two shopping vouchers worth 25 zlotys ($8) each served as the reward for subjects.

#### Materials

To assess individual level of need for closure we used the same version of the Need for Cognitive Closure Scale as in Study 1 (Webster and Kruglanski, 1994) (Cronbach’s α = 0.69^3^). We also used the same manipulation of cognitive load as in Study 1.

##### Information to be acquired preference

In order to measure the dependent variable, we employed a task similar to the classic selective exposure decision paradigm, in which participants were asked to decide whether a manager’s contract should be extended or not (e.g., Frey, 1986; Jonas et al., 2003; Fischer et al., 2005, 2011). Participants were asked to imagine they had just inherited a fashion store. The first decision they needed to take concerned the shop manager’s contract extension. Participants were briefly informed that the manager’s performance over the last year had been a mix of success and failure. The initial information consisted of a balanced number of pieces of information: positive (e.g., “Augustynski introduced a new line of clothes designed for younger customers which turned out to be a success.”) and negative (e.g., “Some of the regular customers were lost because they were not attracted by the new fashion collections.”). Afterward, subjects were given another sheet of paper with 10 two-sentence abstracts of statements about the manager. The number of opinions was also balanced; five were positive (e.g., “He introduced new rules, not everyone likes them but they bring us high premiums. I would like to extend his contract…”) and five were negative (e.g., “Nobody likes to deal with indecisive people. If it were up to me, I would not extend the contract…”). Subjects were informed that every brief statement had the full version (approximately half a page of text) and asked to choose up to five statements which they wanted to receive in the complete form (i.e., found most useful to read in terms of a final decision). They were informed that they would be given a complete version of the chosen opinions. We asked the participants about their initial decision twice: the question “What is your preliminary decision right now?” appeared on both sheets. In order to eliminate results of participants who did not manage to formulate a certain preliminary decision on the basis of the first piece of information (14 people), we analyzed data only for those whose both preliminary decisions were consistent.

The index of preference for expectancy-inconsistent infor mation was defined as the number of selected opinions consistent with the preliminary decision subtracted from the number of selected opinions inconsistent with the preliminary decision. The score was in the range –5 to 5: the bigger the number, the stronger the relative preference for expectancy-inconsistent information.

##### Manipulation check

As in Study 1, we used the two questions to check the effectiveness of cognitive capacity depletion: “How burdensome was the task?” and “How hard was the task?”. Questions were answered on a scale of 1–6 (Cronbach’s α = 0.68). At the end of the study the actual memory of the strings was tested to eliminate participants who did not understand or failed to follow the instructions: the minimum performance was set to at least one correctly recalled character in the correct position. Results of participants who did not meet this criterion (six people) were excluded from the analysis to avoid the risk of analyzing the results of subjects who were not depleted.

#### Procedure

The procedure was similar to that used in Study 1 with the exception of the task used to measure the dependent variable. Participants were informed that they were taking part in a study on business decisions. After obtaining from them informed consent, participants were given instructions printed on paper. Participants were first requested to complete the need for closure questionnaire, after which the subjects were randomly assigned to one of the two conditions. All participants were then given more instructions, and members of the experimental group also received a random sequence of characters to be remembered. Next, subjects gave back their sheets before starting the task measuring the dependent variable. The last part contained a manipulation check questionnaire and a sequence memory test. After collecting all the data, subjects were debriefed and thanked.

### Results and Discussion

#### Manipulation Check and Preliminary Analyses

In the first instance those who changed their initial decision or failed to recall the minimum number of characters in the cognitive load task (16 women, 3 men, 1 gender non-specific) were excluded from further analysis. The mean response for the two questions about the onerousness and difficulty of the task was intended to be used as a manipulation efficiency indicator. Similar to Study 1, the difference between the control (*M* = 2.31, *SD* = 0.95) and experimental (*M* = 2.16, *SD* = 1.00) group was not statistically significant (*t*(72) = 0.622, *p* = 0.54, *d* = 0.15). Again, the manipulation was not effective enough. It was decided to perform the planned analysis, including the manipulation check results in the model as a covariate.

Similarly to Study 1 we checked the results of secondary task itself, they also indicated that the participants performed, on average, very well (*M* = 6.15, *SD* = 2.83), six participants did not recall one single character correctly and was excluded from further analysis.

Neither cognitive capacity depletion (*M*_contr_ = 1.81, *SD* = 2.32, *M*_exp_ = 1.37, *SD* = 2.35, *t*(72) = 0.789, *p* = 0.43, *d* = 0.19) nor need for closure (β = 0.059, *b* = 0.013, *t*(72) = 0.50, *p* = 0.62, 95% CI: [–0.39, 0.65]) affected the preference for expectancy-inconsistent information.

#### The Joint Effect of Need for Closure and Cognitive Capacity on Preference for Expectancy-Inconsistent Questions

**FIGURE 2:**
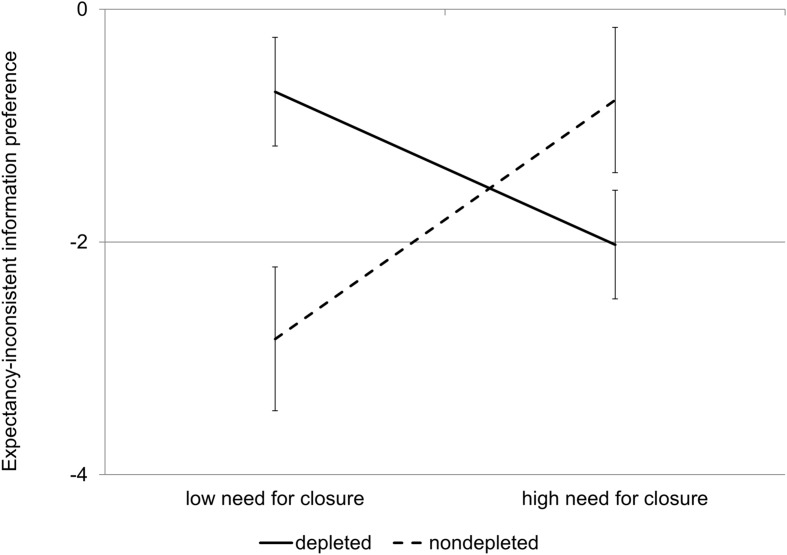
**Interaction effect between need for closure and cognitive capacity on expectancy-inconsistent information preference (Study 2).** Error bars represent standard errors.

We used PROCESS in order to test the interactional effect of need for closure and cognitive capacity depletion on the preference for expectancy-inconsistent information (Hayes, 2013, model 1). The influence of gender and age on the interaction was inspected, but these variables turned out to be statistically insignificant and were removed from the analysis. We found a significant interaction between motivation and cognitive capacity (*b* = –0.16, β = –0.80, *SE* = 0.05, *p* < 0.005, 95% CI [–0.27, –0.05]). The results are shown in **Figure 2.** Further analysis of the interaction showed that need for closure correlated positively with the index of expectancy-inconsistent information preference in the control condition (*b* = –0.097, *β* = –0.99, *SE* = 0.05, *p* = 0.03, 95% CI: [–0.19, –0.01]). In the low capacity condition, the correlation was the opposite and also statistically significant (*b* = 0.06, β = 0.63, *SE* = 0.03, *p* = 0.04, 95% CI: [0.002, 0.122]).

The results of Study 2 confirmed our hypothesis that the pattern of results was identical to that obtained in the previous study. Again it was revealed that in the face of incomplete information, the level of need for closure correlates positively with the preference for expectancy-inconsistent information among high cognitive capacity and negatively among low cognitive capacity subjects. This time the results were even clearer in terms of statistical significance. Additionally, Study 2 provided evidence that incomplete information does not strictly need to mean ‘unanswered questions’ as this time we used brief excerpts from people’s opinions which would be extended when subjects choose them instead of the questions used in Study 1. This modification did not influence the pattern of results.

## General Discussion

The purpose of the present study was to explore the relationship between the need for cognitive closure, cognitive capacity, and the preference for expectancy-inconsistent information as a strategy to reduce uncertainty. According to our predictions, the behavior of individuals confronted with a choice of incomplete information (i.e., questions or partial opinions) was different than when individuals were confronted with complete information (e.g., Dijksterhuis et al., 1996), as happened in previous studies. When confronted with complete pieces of information people high in need for closure tend to focus on those which are expectancy-consistent, but when confronted with incomplete information which is going to be expanded, higher need for closure leads to a stronger preference for expectancy-inconsistent information. Our results are consistent with previous findings showing that the preference for schema-consistent information is not the only possible strategy for achieving closure as in certain situations it may be altered in favor of schema-inconsistent information. Higher need for closure leads to a stronger preference for expectancy-inconsistent information because, in fact, this kind of information does not provide certain details in their original form, however, due to its speculative nature it highlights the lack of knowledge on a given issue which may be easily filled by closer examination of that information (e.g., asking for a complete opinion). Closer examination of the expectancy-inconsistent information may result in twofold alternative consequences: disconfirmation of the suggestion contained in the incomplete information (e.g., negative answer for a question) or acquisition of new and certain information which contradicts an existing preliminary hypothesis, which could potentially undo closure. One could say – and indeed we agree – that this strategy is ‘risky’, but only when an individual’s cognitive capacity is reduced. In this situation, acquisition of contradicting information should result in a weaker feeling of closure, otherwise an individual is able to reconcile this information and therefore minimize the negative impact on closure (Piaget, 1928; Hastie and Kumar, 1979; Crocker et al., 1983; Stangor and McMillan, 1992; Kemmelmeier, 2015). Consequently, we expected that cognitive capacity would play a moderating role in this relationship.

Despite the fact that unanswered questions may be harmful for establishing cognitive closure, finding certain (i.e., contradictory) answers may be also problematic, especially for individuals low in cognitive capacity. The preferred strategy among people striving for certainty is the reconciliation of contradictory information which is effective but also effortful in terms of cognitive capacity (Piaget, 1928; Hastie and Kumar, 1979; Crocker et al., 1983; Stangor and McMillan, 1992; Kemmelmeier, 2015). We therefore expected that only non-depleted individuals would behave in such a way, whereas low cognitive capacity subjects were expected to prefer relatively less expectancy-inconsistent information due to their inability to invest effort in reconciling contradictory information. According to our predictions, cognitive capacity played a moderating role between need for closure and information to be acquired preference. In both studies, higher need for closure caused non-depleted individuals to have a stronger preference for acquiring expectancy-inconsistent information (questions in Study 1 and brief opinions to be extended in Study 2). In contrast, higher need for closure leads to a stronger preference for acquiring expectancy-consistent information among low cognitive capacity subjects.

The main difference between the presented studies and those previously conducted is the informative value of the information utilized used in the main task. While previous studies, with one exception, utilized complete information pieces, we decided to replace them with somehow incomplete information (questions to be asked or brief opinions to be expanded). This provided us with the argument for our expectation that when individuals are instructed to select information to be acquired (e.g., “Which question do you prefer to ask to find out…”), need for closure is linked to stronger relative preference for the expectancy-inconsistent one.

Although the issue of people’s preference for incomplete information in terms of its consistency with previous expectations has not been studied in the last three decades, there is one study which should be addressed here. Kruglanski and Mayseless (1988, Study 2) used a task similar to the one used in Study 1. Subjects were instructed that their task would be to select interview questions they would use to verify the occupation of the target person. The study revealed that subjects under time pressure (which was labeled as “high need for closure condition”) selected relatively less diagnostic questions. One could say that this result challenges our own, however, we believe it does not on the basis of at least two reasons.

Firstly, need for cognitive closure is a complex characteristic (Kruglanski and Webster, 1996; Kruglanski et al., 1997; Kruglanski, 2004) which cannot be fully influenced by time pressure manipulation. Theorists have claimed (e.g., Kruglanski and Webster, 1996; Kruglanski et al., 1997; Kruglanski, 2004) that need for cognitive closure is related to two tendencies: urgency and permanence. The urgency tendency is defined as the “*inclination to ‘seize’ on closure quickly*”, hence it is directly related to haste in the epistemic process. However, the permanence tendency seems to be unrelated to time pressure manipulation since it refers to “*the dual inclination (a) to preserve, or ‘freeze’ on, past knowledge and (b) to safeguard future knowledge*” (Kruglanski and Webster, 1996, p. 265).

Secondly, time pressure manipulation is related not only to motivational consequences, but also the tax on cognitive resources (Roets et al., 2008). It is therefore possible that merely time pressure manipulation (without at least controlling dispositional level of need for closure) may lead to the results with inseparable motivational and cognitive antecedents. To sum up, even though the results of Study 1 and the discussed experiment (Kruglanski and Mayseless, 1988, Study 2) are not compatible with each other, they also do not contradict each other. Nevertheless further investigation on the issue of the combined influence of the dispositional and situationally induced need for closure on the preference for information to be acquired is needed^4^.

The goal of our study was to initialize the investigation on the joint effect of the need for closure and cognitive capacity on the preference for expectancy-inconsistent information which needs to be elaborated. As we expected, the conclusions turned out to be different than the results of analogous studies which analyzed selective focus on complete information. Nevertheless, the two studies presented in this paper are the first step into that interesting area as we have identified two directions which future research should pursue: the consequences of situationally induced need for closure for selection of information to be acquired, and further exploring of the role of cognitive capacity in the discovered relationship.

As we argued above, need for closure understood as a disposition, which we focus on, is one of only two ways of operationalizing this variable. The data provided by Kruglanski and Mayseless (1988) are enough to stipulate that our results address only the dispositional need for cognitive closure. The questions of the interaction between situationally induced need for closure and cognitive capacity remain unanswered. Therefore, we think that future research should investigate the role of situationally induced need for closure with regard to cognitive capacity. Perhaps the exact method of need for closure manipulation will play a role here as, for example, Bukowski et al. (2013) showed that manipulation of need for closure via time pressure has different consequences for an epistemic process than a goal-based one; Bukowski et al. (2013) speculate that the differences may have occurred due to the different consequences of manipulation itself for a cognitive capacity. It is possible that goal-based manipulation of need for closure (e.g., Bukowski et al., 2013; Kossowska and Jaśko, 2013) which does not directly tap cognitive resources will result in a pattern of results similar to the one obtained in the present investigation.

Also the issue of the role of ‘cognitive capacity’ itself needs further exploration. In the current studies cognitive capacity was operationalized narrowly as cognitive load due to a character-repetition task. Since other researchers identified intriguing interactions between need for closure and other objective (e.g., working memory capacity; Kossowska and Jaśko, 2013) or even subjective (e.g., Kossowska and Bar-Tal, 2013) abilities in regard to an epistemic process, it seems interesting to examine whether the effect presented in the current paper is strictly limited to cognitive capacity manipulated by load or, perhaps, will arise also with regard to more general cognitive abilities. According to our interpretation of the results, we expect that every factor which impedes effortful inconsistent information reconciliation should affect the need for closure-information preference relationship in a similar manner.

### Potential Limitations

Some potential limitations of the present studies should be acknowledged. Firstly, the cognitive capacity manipulation yielded a non-significant effect on the manipulation check. Although the absence of a significant manipulation check does not necessarily invalidate an entire study (see, Sigall and Mills, 1998), it should be acknowledged that the results should be treated with caution since they may appear due to other mechanisms. However, as mentioned above, the lack of differences in the manipulation check in the case of cognitive capacity manipulation like the one we used is nothing new (e.g., Bar-Tal et al., 1999, Study 3) and may appear due to the timeline of the study. Hence, given that a similar manipulation yielded significant effects on the manipulation check in several studies (e.g., Roets and Van Hiel, 2007; Roets et al., 2013), we believe the problem is likely to be related to the manipulation check rather than the manipulation itself.

One could say that main effect of need for closure should be expected in a study designed like ours. However, we did not expect the main effects of mere need for closure nor cognitive capacity, because no effects of those variables had been reported previously in studies utilizing a similar method of dependent variable measurement. The main task was based on assessing the usefulness of information pieces, not on cognitive performance whatsoever, thus cognitive capacity was expected to be irrelevant to results. Also the relationship between need for closure and the relative preference for expectancy-inconsistent questions was expected to be null since the memory-based version of the very same task had been used in two previous studies (Bar-Tal and Kossowska, 2010, Study 3; Kossowska and Bar-Tal, 2013, Study 1) and the authors reported no correlation between need for closure and analogous index. This is consistent with the findings of Bar-Tal et al. (2010) that mere epistemic motivation may not be enough to affect information processing style, in fact a certain level of ability may be needed to do that (Bar-Tal et al., 1997, 1999; Bar-Tal and Kossowska, 2010; Kossowska and Bar-Tal, 2013).

The last possible limitation, which was in fact addressed in Study 2, regards the well-known effect of positive–negative asymmetry in social discrimination (Skowronski and Carlston, 1989; Lewicka et al., 1992). In Study 1 we labeled the target person (whose attitude would be verified) as “dishonest” and presented questions concerning morality rather than ability. Thus, the alternative mechanism could be taken into consideration as it was possible that questions concerning negative issues, which are known to be more diagnostic in the moral context, were preferred over those which concerned positive issues. However, Kossowska and Bar-Tal (2013, Study 1) in their study used the very same task, but in addition to ‘dishonest’ used three additional labels (‘honest,’ ‘friendly,’ ‘unfriendly’) in order to check whether the effect of asymmetry would occur. It turned out that the label did not affect information processing in any way. Additionally, in Study 2 we changed the main task, which allowed us to discard this doubt: firstly, this time task did not concern moral issues at all; secondly we did not suggest a preliminary hypothesis; last but not least, the list of brief opinions presented to participants contained information neutral in the context of a moral vs. ability problem (see examples presented above in which only two out of ten information pieces could be interpreted as evaluating ability: “Augustynski has a good relationships with the media” and “Augustynski’s employment has brought financial benefits”). Thus we believe that the issue of positive-negative asymmetry does not apply to the effect presented in the current paper.

## Conclusion

It was generally agreed that higher levels of need for closure are related to expectancy-consistent information preference. However, the present studies show a more complex picture, suggesting that cognitive capacity and the form of information presentation also play a role in addition to epistemic motivation. In particular, it was shown that in the face of incomplete information pieces, people prefer relatively more expectancy-inconsistent ones, which contrasts with previous research on complete information. Furthermore, the key role of cognitive capacity was documented and it turned out that the effect mentioned above takes place only among non-depleted participants, otherwise the relationship is reversed. The present studies highlight the important interplay between motivational and cognitive characteristics on one hand and the form of information presentation on the other, therefore suggesting that the current association between need for closure and rigidity may be an oversimplification as in certain situations individuals who are highly motivated toward closure may also be open to varied information.

## Author Contributions

PS is the author of the main conception and studies design. Performed experiments, gathered, analyzed, and interpreted data. Drafted and prepared the final version of the paper. MK took part in the theoretical basis of the inference framing, critically revised the work, and approved its final version. AS provided substantial contribution to the conception of experiments, critically revised the work and approved its final version and cooperated in preparing and conducting experiments.

## Conflict of Interest Statement

The authors declare that the research was conducted in the absence of any commercial or financial relationships that could be construed as a potential conflict of interest.

## Footnotes

1 By the ‘rule’ the authors understand “inferential devices for categorization, estimation, paired comparisons, and other judgmental tasks that go beyond the information given” (Kruglanski and Gigerenzer, 2011, p. 98), which makes this term close to ways which may be potentially used to formulate the judgment.

2 Results of two participants were excluded. To make sure that this treatment did not affect the results of analyses we performed re-analysis of the data without excluding any of the records. The results obtained this way were similar to those reported below. The only change was in the effect size of the differences between groups in terms of the manipulation check (before the exclusion *d* = 0.07; after the exclusion *d* = 0.19). This difference confirmed concerns about the effectiveness of cognitive capacity depletion of the excluded participants.

3 The Cronbach’s coefficient alpha was acceptable (see Clark and Watson, 1995) and virtually equal in both studies (0.693 in Study 1 and 0.686 in Study 2). Since it turned out to be lower than in the initial study performed during scale adaptation (Kossowska, 2003), we find it important to highlight that the questionnaire was handled according to common practice and all of the reversed questions were transformed prior to analysis.

4 Our postulates on the study of Kruglanski and Mayseless (1988), could not be fulfilled at hat time since the tool assessing the dispositional level of need for closure was designed in early nineties (Webster and Kruglanski, 1994). Thus the most desirable way to ensure our way of reasoning would be replicating our studies with the original task utilized by Kruglanski and Mayseless (1988), however, unfortunately the tool has been lost (personal communication, 26 March, 2015).
